# The effects of capping the alcohol consumption distribution and relative risk functions on the estimated number of deaths attributable to alcohol consumption in the European Union in 2004

**DOI:** 10.1186/1471-2288-13-24

**Published:** 2013-02-18

**Authors:** Gerrit Gmel, Kevin D Shield, Tara AK Kehoe-Chan, Jürgen Rehm

**Affiliations:** 1 Centre for Addiction and Mental Health (CAMH), Toronto, Canada; 2 Faculty of Engineering, University of New South Wales, Sydney, Australia; 3 Institute of Medical Science, University of Toronto, Toronto, Canada; 4 Department of Statistics, University of Toronto, Toronto, Canada; 5 Dalla Lana School of Public Health (DLSPH), University of Toronto, Toronto, Canada; 6 Department of Psychiatry, University of Toronto, Toronto, Canada; 7 Institute for Clinical Psychology and Psychotherapy, TU Dresden, Germany

**Keywords:** Alcohol consumption, Modelling, Gamma distribution, Alcohol-Attributable Fraction, Capping, Mortality, Sensitivity analysis

## Abstract

**Background:**

When calculating the number of deaths attributable to alcohol consumption (i.e., the number of deaths that would not have occurred if everyone was a lifetime abstainer), alcohol consumption is most often modelled using a capped exposure distribution so that the maximum average daily consumption is 150 grams of pure alcohol. However, the effect of capping the exposure distribution on the estimated number of alcohol-attributable deaths has yet to be systematically evaluated. Thus, the aim of this article is to estimate the number of alcohol-attributable deaths by means of a capped and an uncapped gamma distribution and capped and uncapped relative risk functions using data from the European Union (EU) for 2004.

**Methods:**

Sex- and disease-specific alcohol relative risks were obtained from the ongoing Global Burden of Disease, Comparative Risk Assessment Study. Adult *per capita* consumption estimates were obtained from the Global Information System on Alcohol and Health. Data on the prevalence of current drinkers, former drinkers, and lifetime abstainers by sex and age were obtained from various population surveys. Alcohol-attributable deaths were calculated using Alcohol-Attributable Fractions that were calculated using capped (at 150 grams of alcohol) and uncapped alcohol consumption distributions and capped and uncapped relative risk functions.

**Results:**

Alcohol-attributable mortality in the EU may have been underestimated by 25.5% for men and 8.0% for women when using the capped alcohol consumption distribution and relative risk functions, amounting to the potential underestimation of over 23,000 and 1,100 deaths in 2004 in men and women respectively. Capping of the relative risk functions leads to an estimated 9,994 and 468 fewer deaths for men and for women respectively when using an uncapped gamma distribution to model alcohol consumption, accounting for slightly less than half of the potential underestimation.

**Conclusions:**

Although the distribution of drinkers in the population and the exact shape of the relative risk functions at large average daily alcohol consumption levels are not known, the findings of our study stress the importance of conducting further research to focus on exposure and risk in very heavy drinkers.

## Background

Alcohol consumption is one of the most important risk factors for the global burden of disease, and was the third leading cause of Disability Adjusted Life Years lost (burden of disease) globally in 2004 and 2010 [1-3]. Generally, in calculating the number of deaths attributable to alcohol consumption, the most conservative approaches have been used [4,5]. For example, the method used in the 2005/2010 Comparative Risk Analysis (CRA) as part of the Global Burden of Disease (GBD) study to estimate Alcohol-Attributable Fractions (AAFs) (defined as the proportion of deaths that would not have occurred if everyone was a lifetime abstainer; for background on attributable fractions see [6,7] for unadjusted attributable fractions, and [8] for adjusted attributable fractions) assumed that a person could consume a maximum of 150 grams of pure alcohol per day [9]. The approach of capping the continuous alcohol exposure distribution has also been used for country level and regional alcohol-attributable burden of disease studies [10-13].

To mathematically achieve a maximum alcohol consumption cap of 150 grams of alcohol per day, the gamma distribution used to model the consumption of a population of current drinkers is normalized to integrate to 1 when the gamma distribution is integrated from >0 to 150 grams of pure alcohol per day (for a detailed description of this methodology see [14,15]). While data have shown that some people may drink more than 150 grams of alcohol on any given day, it seemed implausible that this level of consumption could be maintained during the biological latency period where alcohol consumption leads to the incidence and development of a disease, and mortality from the disease. However, there is evidence that some people, especially those with alcohol dependence, continue to drink at high levels for prolonged periods of time. For example, a 20-year cohort study of alcohol-dependent patients who had received treatment for their alcoholism found that some participants consumed large average amounts of alcohol over prolonged periods of time. During the times in the cohort study when participants were drinking, average alcohol consumption per day was approximately 140 grams per participant (for a description of the cohort and main results see [16,17]), thus making it probable that the heaviest drinkers consumed above 150 grams of pure alcohol during the biological latency period.

In order to test the effect of capping the alcohol consumption distribution, unsystematic sensitivity analyses that calculated AAFs had been performed for a limited number of alcohol-related diseases [9,14]. These sensitivity analyses indicated that there was not a large difference in the calculated AAFs when alcohol consumption was modelled using a capped or an uncapped distribution. These analyses had been, however, very limited, in that they only addressed breast cancer, pancreatitis and diabetes.

Capping of the gamma distribution changes the nature of the distribution. The domain of the gamma distribution is 0 to ∞; normalization of the gamma distribution changes this domain from 0 to 150 and causes changes to the distribution’s basic characteristics by altering the properties of the shape and scale parameters. Although changes in the gamma distribution after normalization are relatively small, the alteration of the distribution leads to small mathematical inaccuracies [18].

The second conservative assumption generally followed when calculating the number of deaths attributable to alcohol consumption is that the Relative Risks (RRs) do not increase past a large value as consumption increases, with most RR functions being capped after consumption of 120 to 150 grams of alcohol per day; however, in some cases, a lower alcohol consumption limit has been applied to the RR functions [19]). These RR limits were based on the greatest average daily alcohol consumption reported in the underlying cohort studies used in the meta-analyses to calculate the alcohol RR functions. Capping was performed as RR functions obtained from meta-regression are not considered to be valid when consumption is greater than the greatest average daily alcohol consumption reported in these cohort studies (see [20]).

Since the above-noted conservative assumptions have not been previously explored systematically, the aim of our study was to systematically examine the effect of capping the alcohol consumption distribution and the RR functions using alcohol consumption data and mortality data from the EU for 2004.

## Methods

### Exposure estimates

Average daily consumption of alcohol for 2005 (the closest year to 2004 for which data were available) was calculated based on a triangulation of survey data and *per capita* consumption data estimates. Total adult (15 years of age and older) *per capita* consumption of alcohol was calculated by adding the estimates of recorded, unrecorded, and tourist adult *per capita* consumption. All data on recorded (based on official statistics of taxes, sales and/or production, imports and exports), unrecorded (based on population survey data, government monitoring data and expert judgement) and tourist consumption (calculated for countries where there was significant cross border trade (Estonia and Moldova) or countries where the number of tourists that visited a country was greater that the population of the country) were obtained from the Global Information System on Alcohol and Health database (http://apps.who.int/globalatlas/default.asp; see also [21]). The Global Information System collects data via surveys to governments and experts; definitions of the categories described above and the methodology for estimation can be found on the WHO website (http://apps.who.int/globalatlas/default.asp). For 2010 alcohol exposure estimates by country see [22].

### Risk relations

Disease categories where alcohol consumption had a causal impact were identified via standardized methodology [23]. Sources for alcohol-related RR functions by International Classification of Diseases (ICD-10) codes are outlined in Additional file 1: Web Appendix 1. The RR functions and diseases modelled in this paper were based on those in the 2005/2010 CRA for alcohol [3]. The RRs, in most cases, were obtained from meta-analyses reporting a continuous RR function by dose of exposure, i.e., by average daily grams of ethanol consumed (except for tuberculosis and myocardial infarction where categorical risk estimates were used). RRs for current drinkers and former drinkers were obtained from the same meta-analyses (see Additional file 1: Web Appendix 1 for the specific meta-analyses used).

### Relative risk function capping

AAFs were calculated using capped and uncapped RR functions. To prevent unrealistic RR functions, the majority of uncapped RR functions were capped after consumption of 300 grams of alcohol per day, and were changed to linear functions after a consumption of 150 grams of alcohol per day (see Additional file 2: Web Appendix 2). The choice of capping or linearizing the RR function after 150 g/day was made based on the slope of the function at high consumption. If the slope was increasing rapidly, the function was capped right away, if not, it was linearized first.

### Mortality data

Data on the number of deaths by cause, sex, age and country were obtained from the 2004 GBD study [24].

### Alcohol exposure modelling methodology

The prevalence of average daily consumption of alcohol at the population level has been found to be modelled best using the gamma distribution [9]. The gamma distribution has been shown to provide a good fit with alcohol-drinking population data and is very adaptable [9,14]. The gamma distribution can be expressed as follows:

$$\text{Gamma}\left(x,\kappa ,\theta \right)=x^{\kappa -1}\frac{e^{-x/\theta }}{\theta^{\kappa }\Gamma \left(\kappa \right)}\mathrm{for}\;x,\kappa ,\theta >0$$

where κ represents the shape parameter, θ represents the scale parameter, and Γ(κ) is the gamma function and is calculated as follows:

$$\Gamma \left(\kappa \right)=\int_{>0}^{\infty }t^{\kappa -1}e^{-t}\mathrm{dt}$$

The gamma distribution displays some properties that make it easily manageable. For example:

$$\theta =\frac{\sigma^{2}}{\mu }\;\text{and}\;\kappa =\frac{\mu^{2}}{\sigma^{2}}$$

Also, the mean of the gamma distribution is equal to the empirical mean.

Previous research examining the relationship between mean alcohol consumption and the standard deviation of the gamma distribution using data from 851 datasets found a strong linear relationship between the mean and the standard deviation of the alcohol consumption gamma distribution [9]. The standard deviation of the gamma distribution for any population (and any age group within a population) can be calculated as follows:

For men:

$$\sigma_{\text{men}}=1.171\cdot \mu_{\text{men}}$$

For women:

$$\sigma_{\text{women}}=1.258\cdot \mu_{\text{women}}$$

Thus, only data on adult *per capita* consumption and the prevalence of current drinkers are needed in order to model the alcohol consumption distribution [5,9,14]. To account for alcohol that is not consumed due to spillage, breakage and waste, and undercoverage in the medical epidemiology studies that are used to calculate alcohol RRs, 80% of total *per capita* consumption is used when empirically modelling alcohol consumption using the gamma distribution (for a detailed discussion see [5]).

The alcohol consumption distribution cap of 150 grams per day was implemented by normalizing the gamma distribution, so that when consumption was integrated from >0 to 150 grams of alcohol per day, the integral would be equal to 1. It should be noted that the use of the notation *>*0 indicates the exclusion of 0 in the integration, as an average consumption of 0 grams per day does not qualify a person as a drinker. This, however, leaves no mathematical artifact, as the gamma distribution at 0 always equals 0.

### Modelling alcohol-attributable deaths

The AAFs were calculated for the total number of deaths attributable to alcohol consumption according to CRA methodology [3], and, thus, calculated the number of deaths that would not be present under the counterfactual scenario that everyone was a lifetime abstainer (thus, lifetime abstainers were used as the reference category for risks). The decision to use lifetime abstention as the reference follows the earlier CRAs for alcohol (see [3,5]; see [25] for a detailed discussion of this choice). The AAFs were calculated as follows (using the cap at 150 g/day):

$$\text{AAF}=\frac{P_{\text{abstainer}}RR_{\text{abstainer}}+P_{\text{former}}RR_{\text{former}}+\int_{>0}^{150}P_{\text{current}}\left(x\right)RR_{\text{current}}\left(x\right)\text{dx}-1}{P_{\text{abstainer}}RR_{\text{abstainer}}+P_{\text{former}}RR_{\text{former}}+\int_{>0}^{150}P_{\text{current}}\left(x\right)RR_{\text{current}}\left(x\right)\mathrm{dx}}$$

where P_abstainer_ represents the prevalence of lifetime abstainers, RR_abstainer_ represents the relative risk for lifetime abstainers (equals 1 as lifetime abstainers are the reference group), P_former_ represents the prevalence of former drinkers, P_current_(x) represents the prevalence of current drinkers who on average consume × grams of alcohol per day, RR_former_ is the relative risk for a disease for a former drinker, and RR_current_(x) is the relative risk for a disease for a current drinker who on average drinks × grams of alcohol per day. In the uncapped scenario, the integral would be evaluated between 0 and infinity. The separation between lifetime abstainers and former drinkers was based on the different associated risks. Former drinkers usually have higher risks for disease and mortality (see [23] for an overview of RRs for former drinkers for different disease categories), as a substantial number of former drinkers have quit drinking for health reasons (see the so-called sick quitter hypotheses, e.g., [26,27]).

The above attributable fraction is based on continuous exposure and risk functions. Such an approach is deemed, in general, to be advantageous compared to categorical distributions [28,29] because it involves no loss of information. However, for alcohol, the difference between capped distributions and the categorical approach was not large [14]. In some sense, capping of RRs introduces an element of the categorical approach into the continuous approach.

### Normalization

To assess the changes in the AAFs due to the normalization of the gamma function, we compared the estimated number of alcohol-attributable deaths in the EU for 2004 obtained with a normalized prevalence distribution (from >0 to 150 grams per day) with those obtained with a non-normalized prevalence distribution (from >0 to infinity). To depict the difference in normalization at higher levels of consumption, we compared the normalized and non-normalized gamma distributions for men 15 years of age and older in Latvia; Latvian men were chosen as the relatively high alcohol consumption levels in Latvia make the difference between the capped and uncapped distributions more salient.

## Results

**Figure 1:**
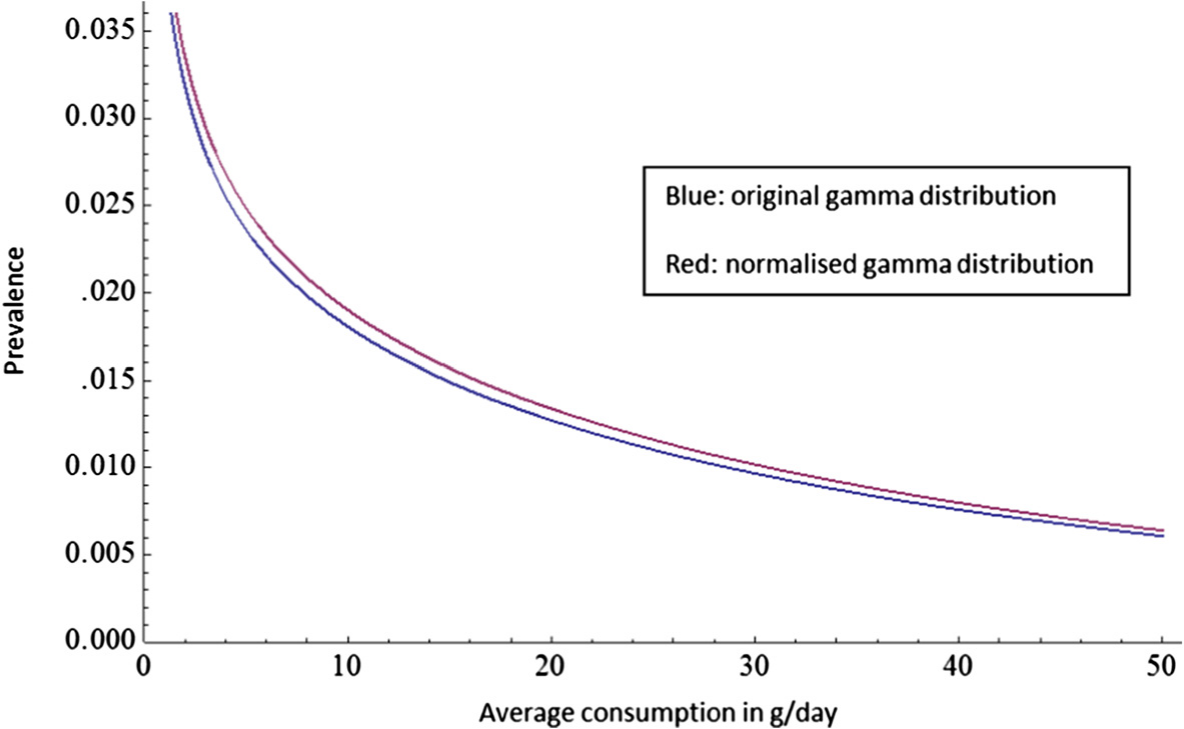
Original and normalized gamma distributions for men in Latvia.

Figure 1 shows the original and the normalized gamma distributions for men 15 years of age and older in Latvia. When the gamma distribution is normalized, we observed a higher average daily consumption of alcohol among current drinkers, resulting in a greater change in the alcohol consumption distribution when normalized. However, as displayed in Figure 1, for men aged 15 years and older in Latvia, the effect of this shift is minimal. As Latvia has the highest average consumption among current drinkers in the EU (consumption was 35.8 litres per current drinker for 2005), the shift in the consumption distribution would be less than that for other EU countries for 2005.

Table 1 displays the prevalence of average daily alcohol consumption among current drinkers of >0 to <150 grams per day, 150 to <200 grams per day, and 200+ grams per day for the EU in 2005 using an unadjusted alcohol consumption distribution. These numbers stem from an gamma distribution that was based on the aggregated alcohol consumption from all countries in the EU (see also [30], for details on country distributions). For women, we found that 99.99% of current drinkers consumed less than 150 grams of alcohol per day and 0.001% consumed between 150 and <200 grams per day. In the EU in 2005, 98.30% of male current drinkers consumed between >0 and <150 grams of alcohol per day, 1.20% consumed between 150 and <200 grams per day, and 0.50% consumed 200 grams or more of alcohol per day. The shift in the mean alcohol consumption is negligible for women. For men, the normalization of the gamma distribution to 150 grams per day leads to a difference in mean alcohol consumption downwards of 2.85 grams per day (from 31.97 to 29.12 g/day).

**Table 1:** Prevalence of average daily alcohol consumption in the European Union

**Average daily consumption in grams per day**	**Females**	**Males**
>0 to <150	99.99%	98.30%
150 to <200	0.01%	1.20%
200 grams and greater	0.00%	0.50%

Table 2 outlines the AAFs for men and women in the EU for 2005 by disease category with the alcohol consumption gamma distribution capped and uncapped, while keeping the RR functions capped. Table 3 outlines the AAFs using uncapped RR functions. The change in the AAFs when capping the alcohol consumption distribution and/or the RR functions is greater for men than for women. This simply stems from the fact that fewer women than men drink 150 grams or more of alcohol per day.

**Table 2:** AAFs for males and females in the European Union, using a capped and an uncapped gamma distribution, and with capped relative risk functions

	**Females**		**Males**	
**Cause of death**	**Capped**	**Uncapped**	**Capped**	**Uncapped**
Oral cavity and pharynx cancer	26.3%	26.3%	53.0%	55.5%
Esophageal cancer	15.1%	15.2%	34.8%	39.4%
Colorectal cancer	5.2%	5.2%	7.9%	8.7%
Liver cancer	9.0%	9.0%	17.5%	18.7%
Breast cancer	10.8%	10.8%	0.0%	0.0%
Epilepsy	14.7%	14.7%	33.5%	40.5%
Lower respiratory infections	7.0%	7.0%	13.2%	14.8%
Stroke	−1.1%	−0.9%	10.4%	12.8%
Hypertension	8.0%	8.3%	23.9%	28.0%
Liver cirrhosis	49.3%	49.9%	71.7%	85.2%
Diabetes	−6.4%	−6.2%	−1.4%	4.2%
Tuberculosis	10.3%	10.3%	31.7%	32.7%
Ischemic heart disease	−7.2%	−7.2%	−11.2%	−11.0%
Motor vehicle accidents	9.0%	9.1%	14.2%	14.7%
Suicide	9.0%	9.1%	14.2%	14.7%
Other injuries	9.0%	9.1%	14.2%	14.7%

**Table 3:** AAFs for males and females in the European Union, using a capped and an uncapped gamma distribution, and with uncapped relative risk functions

	**Females**		**Males**	
**Cause of death**	**Capped**	**Uncapped**	**Capped**	**Uncapped**
Oral cavity and pharynx cancer	26.3%	26.3%	53.0%	55.8%
Esophageal cancer	15.1%	15.2%	34.8%	41.2%
Colorectal cancer	5.2%	5.2%	7.9%	9.0%
Liver cancer	9.0%	9.0%	17.5%	18.9%
Breast cancer	10.8%	10.8%	0.0%	0.0%
Epilepsy	14.7%	14.7%	33.5%	44.8%
Lower respiratory infections	7.0%	7.0%	13.2%	15.4%
Stroke	−1.1%	−0.8%	10.4%	13.8%
Hypertension	8.0%	8.4%	23.9%	30.1%
Liver cirrhosis	49.3%	50.2%	71.7%	93.4%
Diabetes	−6.4%	−6.2%	−1.4%	25.3%
Tuberculosis	10.3%	10.3%	31.7%	32.7%
Ischemic heart disease	−7.2%	−7.2%	−11.2%	−11.0%
Motor vehicle accidents	9.0%	9.1%	14.2%	14.7%
Suicide	9.0%	9.1%	14.2%	14.7%
Other injuries	9.0%	9.1%	14.2%	14.7%

Table 4 outlines the number of deaths by cause and sex that were attributable to alcohol using a capped and an uncapped alcohol consumption distribution and an uncapped RR function. We observed that 24,242 fewer alcohol-attributable deaths were estimated when the alcohol consumption distribution and the RR functions were capped. Of the 24,242 alcohol-attributable deaths, 95% were in men. This represents a relative difference of 23.1% (25.5% for men and 8.0% for women) in the number of deaths as compared to the uncapped distribution.

**Table 4:** Alcohol-attributable deaths for people aged 15 years and older in the European Union calculated using capped (at 150 grams per day) and uncapped alcohol consumption distributions and uncapped relative risk functions

	**Females**		**Males**	
**Cause of death**	**Capped**	**Uncapped**	**Capped**	**Uncapped**
Oral cavity and pharynx cancer	1,719	1,721	12,935	13,563
Esophageal cancer	1,235	1,238	8,492	9,632
Colorectal cancer	4,008	4,012	6,884	7,570
Liver cancer	1,459	1,460	5,259	5,619
Breast cancer	11,048	11,073	0	0
Epilepsy	472	473	1,650	1,993
Lower respiratory infections	5,297	5,304	8,171	9,138
Stroke	−3,507	−2,895	22,803	28,097
Hypertension	5,193	5,396	9,285	10,878
Liver cirrhosis	13,840	14,019	41,721	49,549
Diabetes	−3,995	−3,893	−637	1,877
Tuberculosis	238	238	1,830	1,889
Ischemic heart disease	−30,117	−30,113	−50,684	−49,698
Motor vehicle accidents	1,067	1,067	5,386	5,551
Suicide	1,379	1,380	6,990	7,203
Other injuries	5,006	5,008	10,467	10,787
Total deaths	14,342	15,488	90,552	113,648

Table 5 outlines the number of deaths by cause and sex attributable to alcohol consumption using a capped and an uncapped alcohol consumption distribution and capped RR functions. We can conclude that of the 24,242 difference in alcohol-attributable deaths that comes from capping the alcohol consumption distribution and the RR functions, 43.2% (43.3% for men and 40.8% for women) is due to capping of the RR function. The majority of the difference in mortality observed when comparing the capped and uncapped RR functions (while keeping the alcohol consumption distribution uncapped) was due to deaths from liver cirrhosis. When comparing the resulting AAFs and deaths for men and women estimated using a capped and an uncapped gamma distribution, there was a greater protective effect of alcohol consumption observed for the capped distribution when compared to the uncapped distribution (although some estimates seem to be equal, the capped estimates are lower; however, the difference is not shown in the number of digits presented for these estimates).

**Table 5:** Alcohol-attributable deaths for people aged 15 years and older in the European Union calculated using capped (at 150 grams per day) and uncapped alcohol consumption distributions and capped relative risk functions

	**Females**		**Males**	
**Cause of death**	**Capped**	**Uncapped**	**Capped**	**Uncapped**
Oral cavity and pharynx cancer	1,719	1,721	12,935	13,499
Esophageal cancer	1,235	1,238	8,492	9,206
Colorectal cancer	4,008	4,011	6,884	7,351
Liver cancer	1,459	1,460	5,259	5,553
Breast cancer	11,048	11,068	0	0
Epilepsy	472	473	1,650	1,802
Lower respiratory infections	5,297	5,303	8,171	8,790
Stroke	−3,507	−3,189	22,803	26,067
Hypertension	5,192	5,322	9,285	10,129
Liver cirrhosis	13,840	13,926	41,721	45,213
Diabetes	−3,995	−3,893	−637	312
Tuberculosis	238	238	1,830	1,889
Ischemic heart disease	−30,117	−30,113	−50,684	−49,698
Motor vehicle accidents	1,067	1,067	5,386	5,551
Suicide	1,379	1,380	6,990	7,203
Other injuries	5,006	5,008	10,467	10,787
Total deaths	14,341	15,020	90,552	103,654

## Discussion

The results of our study indicate that for the EU in 2004 there was a substantial difference in the number of estimated alcohol-attributable deaths when either the alcohol consumption distribution or the RR functions are capped. However, it is uncertain if capping leads to better estimates of the actual number of alcohol-attributable deaths as literature on very heavy drinkers and their resulting increased risk of alcohol-related diseases is scarce. In alcohol epidemiology, cohorts that are used to measure the underlying risk relationships are usually selected to maximize the follow-up rate, and, thus, these cohorts are typically composed of special populations with comparatively few heavy and virtually no very heavy drinkers [20,31,32]. Even in cohorts that are representative of the general population, many heavy or very heavy drinkers are excluded by study design, as they are not typically included in the sampling frame (which often excludes homeless/institutionalized individuals) or are less likely to respond and participate in studies when contacted [13].

Studies on the risk of mortality from alcohol-related diseases for people with alcohol dependence (who consume a large amount of alcohol) do exist, and show a high RR of mortality for this population; however, these studies do not provide the underlying alcohol consumption for this population and, thus, it is impossible to rely on these studies to improve the RR functions at high alcohol consumption levels [30,33,34]. Moreover, it is not clear if alcohol consumption or some co-morbid conditions associated with alcohol dependence were responsible for the high mortality observed in these studies.

Given the uncertainties outlined above, research on the morbidity and mortality consequences of heavy and very heavy drinking is urgently needed to adequately assess the burden of disease attributable to alcohol consumption. The results of these required studies ideally will determine whether the high consumption levels observed in studies of individuals with alcohol dependence can, in fact, be sustained over long periods of time, and will determine the risk relationships between very heavy drinking and different disease categories (for a first attempt at estimating these risk relationships see [33]).

The estimated number of alcohol-attributable deaths due to liver cirrhosis was the category that was most affected by the capping of the RR functions. Liver cirrhosis, in particular, is associated with longer term heavy drinking, with some studies finding that overall exposure to alcohol is the most important factor in developing this disease [35,36]. However, the exact form of the risk function for very heavy drinkers is still unknown, as is whether the RR function for liver cirrhosis in fact continues to increase exponentially with increasing exposure at very high alcohol consumption levels. Capping the gamma distribution also resulted in a greater estimated effect of alcohol consumption on ischemic heart disease, stroke and diabetes. This greater protective effect was due to the normalization of the gamma distribution resulting in a greater prevalence of moderate drinkers (drinkers who experience a protective effect for the aforementioned diseases) when compared to the uncapped distribution. These results contrast with earlier non-systematic analyses of the effects of capping which showed very little difference between the AAF estimates using capped and uncapped models [9]. This contrast is easily explained when considering that the diseases that make up most of the difference have not been studied in previous analyses. In the previous analyses, Kehoe and colleagues only analysed the effects of capping the gamma distribution at 300 grams per day (where most studies have used a cap of 150 grams per day) and only looked at the effect of capping the gamma distribution of the estimated AAF for diabetes, breast cancer and pancreatitis [9].

Capping seems to be potentially problematic only in the male population, due to the larger proportion of men, as compared to women, who drink over 150 grams of alcohol per day. The 150 g/day cap is not sex specific. This lack of a sex specific cap creates a problem as men with alcohol use disorders (heavy consumers of alcohol) usually consume more alcohol than do women with alcohol use disorders [37]. Thus, the 150 g/day cap theoretically leads to a larger bias when estimating the alcohol-attributable burden of disease for men as compared to the bias the cap theoretically leads to when estimating the alcohol-attributable burden of disease for women. It could be argued that gender specific capping could lead to more accurate results; however, as mentioned previously, RR data for sustained very high alcohol consumption are largely unavailable, which is an issue that should also be addressed.

This study has limitations in terms of the data used to compare the estimated alcohol-attributable mortality when using a capped and an uncapped alcohol consumption distribution. Specifically, this paper was limited by the absence of age-specific estimates for the EU due to a lack of age-specific reported information on the prevalence of current drinkers, former drinkers, and lifetime abstainers, and the relative amount of alcohol consumed. Additionally, data on the uncertainty of the RR functions after 150 g/day were in most cases not available, and, thus, it was impossible to calculate the 95% confidence intervals for the burden of disease attributable to alcohol consumption. The limitations of this study which are inherent to all burden of alcohol studies include inaccuracies with alcohol exposure data, risk estimates (discussed previously), and mortality data. The accuracy of the measurement of lifetime abstainers in a population and in the observational studies that were used in the meta-analyses that the RR estimates were based upon are subject to multiple biases and often lifetime abstainers are misclassified as former drinkers [38]. Alcohol drinking status estimates are also limited by the biases introduced by the surveys that measure them [13]. The alcohol consumption estimates (i.e. per capita consumption of alcohol) are subject to random error with data on unrecorded adult per capita consumption and tourist per capita consumption having substantially more uncertainty than does recorded consumption [39]. Thus for countries as recorded adult per capita consumption and tourist per capita consumption proportionally make up more of total adult per capita consumption the larger the random error will be for the measured total adult per capita consumption. Additionally, the categorization of deaths by cause is often miscoded or coded in junk categories [40,41]. Although these biases will affect the measured difference in mortality when using a capped and an uncapped alcohol consumption distribution, this paper demonstrates that there is a need to determine an accurate cap as there is a large difference between the calculated alcohol-attributable mortality when using a capped and an uncapped distribution. As this analysis does not assess the goodness of fit of alcohol consumption distribution models and does not adjust for any confounding factors, the numbers presented in this manuscript should not be taken as “true” estimates as they are only used to demonstrate the effect of capping the alcohol consumption distribution on the estimated alcohol-attributable mortality.

Future research should focus on determining an accurate alcohol consumption cap rather than assessing the impact of these biases on the differences between uncapped and capped alcohol consumption distributions; once an accurate alcohol consumption cap is determined, it should always be used.

## Conclusion

Capping of the alcohol consumption distribution or of the RR functions is a conservative approach when estimating the number of deaths attributable to alcohol consumption and may lead to an underestimation of alcohol-attributable mortality. Further research is necessary to determine the extent to which the capping of alcohol consumption and RR functions leads to an underestimation of the actual number of alcohol-attributable deaths.

## Abbreviations

AAF: Alcohol-Attributable Fraction; GBD: Global Burden of Disease; EU: European Union; RR: Relative Risk.

## Competing interests

The authors declare that they have no competing interests.

## Authors’ contributions

GG performed the majority of the statistical analyses and took the lead in preparing the manuscript. KS helped to design the study, performed some of the analyses, and contributed to the preparation of the manuscript. TK-C helped to design the study and wrote portions of the programming for the statistical tests. JR designed the study, supervised all aspects of the work, and contributed to the preparation of the manuscript. All authors read and approved the final manuscript.

## Pre-publication history

The pre-publication history for this paper can be accessed here:

http://www.biomedcentral.com/1471-2288/13/24/prepub

## Supplementary Material

Additional file 1 **Web-appendix 1.** Sources for relative risk functions. Click here for file

Additional file 2 **Web-appendix 2.** Examples of extrapolated behaviour of relative risk functions after consumption of 150 grams of alcohol per day. Click here for file
