# Supplementary material for: The effects of capping the alcohol consumption distribution and relative risk functions on the estimated number of deaths attributable to alcohol consumption in the European Union in 2004
Source: BMC Med Res Methodol. 2013 Feb 18;13:24. doi: 10.1186/1471-2288-13-24 (PMC3584740; doi:10.1186/1471-2288-13-24)
Supplement: Additional file 1 — Web-appendix 1. Sources for relative risk functions. [file 1471-2288-13-24-S1.docx]

## Appendix 1. Categories of alcohol-related disease and sources used for determining alcohol-attributable fractions

| **Condition** | | **ICD 10 Code** | **Source for AAF** |
| --- | --- | --- | --- |
| **Infectious and parasitic diseases** | |  |  |
|  | Tuberculosis | A15-A19 | (Lönnroth et al., 2008); for causal relationship see: (Rehm et al., 2009) |
| Human immunodeficiency virus/ Acquired immune deficiency syndrome | | B20-B24 | (Gmel et al., 2011) |
| **Malignant neoplasm's** | |  |  |
|  | Mouth and oropharynx cancers | C00-C14 | (Baan et al., 2007; International Agency for Research on Cancer, 2011) (based on Relative Risks from (Corrao et al., 2004)) |
|  | Esophageal cancer | C15 | (Baan et al., 2007; International Agency for Research on Cancer, 2011) (based on Relative Risks from (Corrao et al., 2004)) |
|  | Liver cancer | C22 | (Baan et al., 2007; International Agency for Research on Cancer, 2011) (based on Relative Risks from (Corrao et al., 2004)) |
|  | Laryngeal cancer | C32 | (Baan et al., 2007; International Agency for Research on Cancer, 2011) (based on Relative Risks from (Corrao et al., 2004)) |
|  | Breast cancer | C50 | (Baan et al., 2007; International Agency for Research on Cancer, 2011) (based on Relative Risks from (Corrao et al., 2004)) |
|  | Colon cancer | C18 | (Baan et al., 2007; International Agency for Research on Cancer, 2011) (based on Relative Risks from (Corrao et al., 2004)) |
|  | Rectal cancer | C20 | (Baan et al., 2007; International Agency for Research on Cancer, 2011) (based on Relative Risks from (Corrao et al., 2004)) |
| **Diabetes** | |  |  |
|  | Diabetes mellitus | E10-E14 | (Baliunas et al., 2009) |
| **Neuro-psychiatric conditions** | |  |  |
|  | Alcoholic psychoses (part of AUD) | F10.0, F10.3-F10.9 | 100% AAF per definition |
|  | Alcohol abuse (part of AUD) | F10.1 | 100% AAF per definition |
|  | Alcohol dependence (part of AUD) | F10.2 | 100% AAF per definition |
|  | Epilepsy | G40-G41 | (Samokhvalov et al., 2010a) |
| **Cardiovascular disease** | |  |  |
|  | Hypertensive disease | I10-I15 | (Taylor et al., 2009) |
|  | Ischemic heart disease | I20-I25 | (Roerecke & Rehm, 2012), for volume, (Roerecke & Rehm, 2010) for pattern |
|  | Cardiac arrhythmias | I47-I49 | (Samokhvalov et al., 2010b) |
|  | Ischemic stroke | I63-I66 | (Patra et al., 2010) |
|  | Hemorrhagic and other non-ischemic stroke | I60-I62 | (Patra et al., 2010) |
| **Digestive diseases** | |  |  |
|  | Cirrhosis of the liver | K70, K74 | (Rehm et al., 2010) |
|  | Acute and chronic pancreatitis | K85, K86.1 | (Irving et al., 2009) |
| **Respiratory infections** | |  |  |
|  | Pneumonia | J10.0, J11.0, J12-J15, J18 | (Samokhvalov et al., 2010c) |
| **Conditions arising during the prenatal period** | |  |  |
|  | Low birth weight: as defined by the global burden of disease | P05-P07 | (Patra et al., 2011) |
| **Unintentional injuries** | |  |  |
|  | Motor vehicle accidents | § | (Taylor et al., 2010) for Relative Risk, methodology adopted from (Taylor et al., 2011) |
|  | Poisonings | X40-X49 | (Taylor et al., 2010) for Relative Risk, methodology adopted from (Taylor et al., 2011) |
|  | Falls | W00-W19 | (Taylor et al., 2010) for Relative Risk, methodology adopted from (Taylor et al., 2011) |
|  | Fires | X00-X09 | (Taylor et al., 2010) for Relative Risk, methodology adopted from (Taylor et al., 2011) |
|  | Drowning | W65-W74 | (Taylor et al., 2010) Relative Risk, methodology adopted from (Taylor et al., 2011) |
|  | Other Unintentional injuries | †Rest of V-series and W20-W64, W 75-W99, X10-X39, X50-X59, Y40-Y86, Y88, and Y89 | (Taylor et al., 2010) for Relative Risk, methodology adopted from (Taylor et al., 2011) |
| **Intentional injuries** | |  | (Taylor et al., 2010) for Relative Risk, methodology adopted from (Taylor et al., 2011) |
|  | Self-inflicted injuries | X60-X84 and Y87.0 | (Taylor et al., 2010) for Relative Risk, methodology adopted from (Taylor et al., 2011) |
|  | Homicide | X85-Y09, Y87.1 | (Taylor et al., 2010) for Relative Risk, methodology adopted from (Taylor et al., 2011) |
|  | Other intentional injuries |  | (Taylor et al., 2010) for Relative Risk, methodology adopted from (Taylor et al., 2011) |
| § V021–V029, V031–V039, V041–V049, V092, V093, V123–V129, V133–V139, V143–V149, V194–V196, V203–V209, V213–V219, V223–V229, V233–V239, V243–V249,V253–V259, V263–V269, V273– V279, V283–V289, V294–V299, V304–V309, V314–V319, V324–V329, V334–V339, V344–V349, V354–V359, V364–V369, V374–V379, V384–V389, V394–V399, V404–V409, V414–V419, V424–V429, V434–V439, V444–V449, V454–V459, V464– V469, V474–V479, V484–V489, V494–V499, V504–V509, V514–V519, V524–V529, V534–V539, V544–V549, V554–V559, V564–V569, V574–V579, V584–V589, V594–V599, V604–V609, V614–V619, V624–V629, V634–V639, V644–V649, V654– V659, V664–V669, V674–V679, V684–V689, V694–V699, V704–V709, V714–V719, V724–V729, V734–V739, V744–V749, V754–V759, V764–V769, V774–V779, V784–V789, V794–V799, V803–V805, V811, V821, V830–V833, V840–V843, V850– V853, V860–V863, V870–V878, V892. †Rest of V = V-series MINUS §. | | | |

References

Baan, R, Straif, K, Grosse, Y, Secretan, B, El Ghissassi, F, Bouvard, V, Alteri, A, Cogliano, V, On behalf of the WHO International Agency for Research on Cancer monograph working group. (2007). Carcinogenicity of alcoholic beverages. *Lancet Oncol* **8:** 292-293

Baliunas, D, Taylor, B, Irving, H, Roerecke, M, Patra, J, Mohapatra, S, Rehm, J. (2009). Alcohol as a risk factor for type 2 diabetes - A systematic review and meta-analysis. *Diabetes Care* **32:** 2123-2132

Corrao, G, Bagnardi, V, Zambon, A, La Vecchia, C. (2004). A meta-analysis of alcohol consumption and the risk of 15 diseases. *Prev Med* **38:** 613-619

Gmel, G, Shield, K, Rehm, J. (2011). Developing a methodology to derive alcohol-attributable fractions for HIV/AIDS mortality based on alcohol's impact on adherence to antiretroviral medication. *Popul Health Metr* **9:** 5

International Agency for Research on Cancer. (2011). IARC Monograph 96 on the Evaluation of Carcinogenic Risks to Humans. Alcoholic beverage consumption and ethyl carbamate (urethane). Lyon, France: International Agency for Research on Cancer (IARC).

Irving, HM, Samokhvalov, A, Rehm, J. (2009). Alcohol as a risk factor for pancreatitis. A systematic review and meta-analysis. *JOP* **10:** 387-392

Lönnroth, K, Williams, B, Stadlin, S, Jaramillo, E, Dye, C. (2008). Alcohol use as a risk factor for tuberculosis - a systematic review. *BMC Public Health* **8:** 289

Patra, J, Taylor, B, Irving, H, Roerecke, M, Baliunas, D, Mohapatra, S, Rehm, J. (2010). Alcohol consumption and the risk of morbidity and mortality from different stroke types - a systematic review and meta-analysis. *BMC Public Health* **10:** 258

Patra, J, Bakker, R, Irving, H, Jaddoe, VWV, Malini, S, Rehm, J. (2011). Dose-response relationship between alcohol consumption before and during pregnancy and the risks of low birthweight, preterm birth and small for gestational age (SGA)-a systematic review and meta-analyses. *BJOG: International Journal of Obstetrics and Gynaecology* **118:** 1411-1421 10.1111/j.1471-0528.2011.03050.x.

Rehm, J, Kehoe, T, Gmel, G, Stinson, F, Grant, B, Gmel, G. (2010). Statistical modeling of volume of alcohol exposure for epidemiological studies of population health: the example of the US. *Popul Health Metr* **8:** 3

Rehm, J, Mathers, C, Popova, S, Thavorncharoensap, M, Teerawattananon, Y, Patra, J. (2009). Global burden of disease and injury and economic cost attributable to alcohol use and alcohol use disorders. *Lancet* **373:** 2223-2233

Roerecke, M, Rehm, J. (2010). Irregular heavy drinking occasions and risk of ischemic heart disease: a systematic review and meta-analysis. *Am J Epidemiol* **171:** 633-644

Roerecke, M, Rehm, J. (2012). The cardioprotective association of average alcohol consumption and ischaemic heart disease: a systematic review and meta-analysis. *Addiction*

Samokhvalov, AV, Irving, H, Mohapatra, S, Rehm, J. (2010a). Alcohol consumption, unprovoked seizures and epilepsy: a systematic review and meta-analysis. *Epilepsia* **51:** 1177-1184 Doi:10.1111/j.1528-1167.2009.02426.x.

Samokhvalov, AV, Irving, HM, Rehm, J. (2010b). Alcohol as a risk factor for atrial fibrillation: a systematic review and meta-analysis. *Eur J Cardiovasc Prev Rehabil* **17:** 706-712

Samokhvalov, AV, Irving, HM, Rehm, J. (2010c). Alcohol consumption as a risk factor for pneumonia: systematic review and meta-analysis. *Epidemiol Infect* **138:** 1789-1795

Taylor, B, Irving, HM, Baliunas, D, Roerecke, M, Patra, J, Mohapatra, S, Rehm, J. (2009). Alcohol and hypertension: gender differences in dose-response relationships determined through systematic review and meta-analysis. *Addiction* **104:** 1981-1990

Taylor, B, Irving, HM, Kanteres, F, Room, R, Borges, G, Cherpitel, C, Greenfield, T, Rehm, J. (2010). The more you drink, the harder you fall: a systematic review and meta-analysis of how acute alcohol consumption and injury or collision risk increase together. *Drug Alcohol Depend* **110:** 108-116 DOI: 10.1016/j.drugalcdep.2010.02.011.

Taylor, B, Shield, K, Rehm, J. (2011). Combining best evidence: A novel method to calculate the alcohol-attributable fraction and its variance for injury mortality. *BMC Public Health* **11:** 265
