# Supplementary material for: The effects of capping the alcohol consumption distribution and relative risk functions on the estimated number of deaths attributable to alcohol consumption in the European Union in 2004
Source: BMC Med Res Methodol. 2013 Feb 18;13:24. doi: 10.1186/1471-2288-13-24 (PMC3584740; doi:10.1186/1471-2288-13-24)
Supplement: Additional file 2 — Web-appendix 2. Examples of extrapolated behaviour of relative risk functions after consumption of 150 grams of alcohol per day. [file 1471-2288-13-24-S2.docx]

## Web-appendix 2: Examples of extrapolated behaviour of relative risk functions after 150 grams per day.


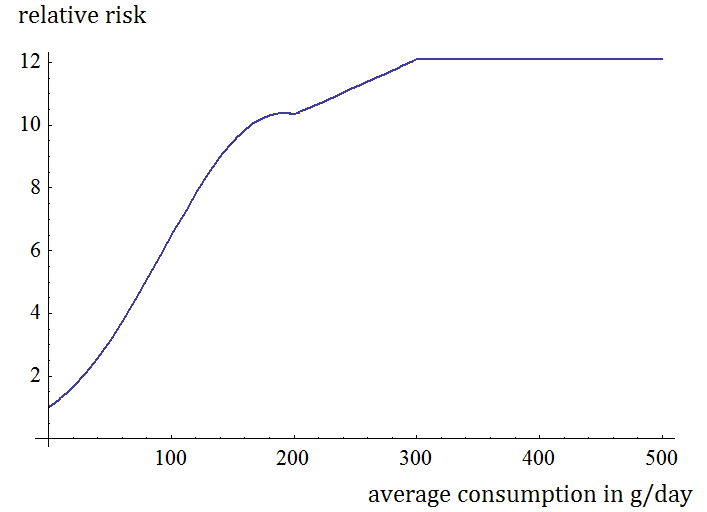


Figure 1: relative risk function for oral cavity and pharynx cancer

In the case of Oral Cavity and Pharynx cancer, the behaviour of the risk function for a daily alcohol intake larger than 200g/day was a piecewise linear function. Between 200g/day and 300g/day, the function is linear with a slope equal to the average slope of the function between 150g/day and 200g/day. After 300g/day, the function is simply kept constant. A similar shape has been adopted for liver cirrhosis.


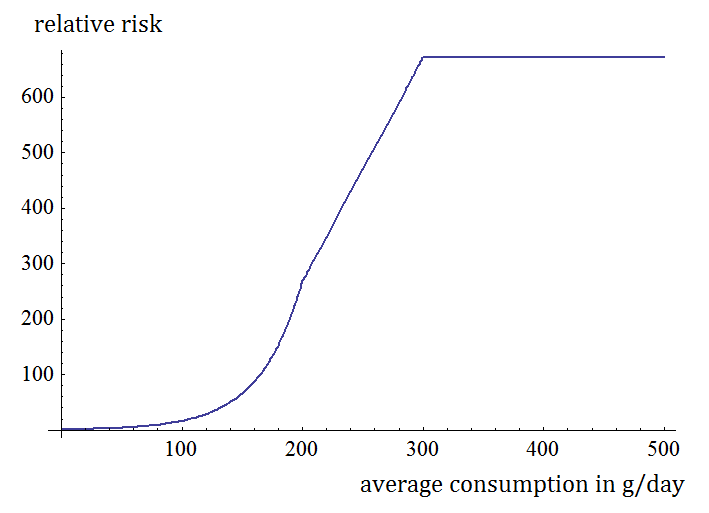


Figure 2: relative risk function of liver cirrhosis


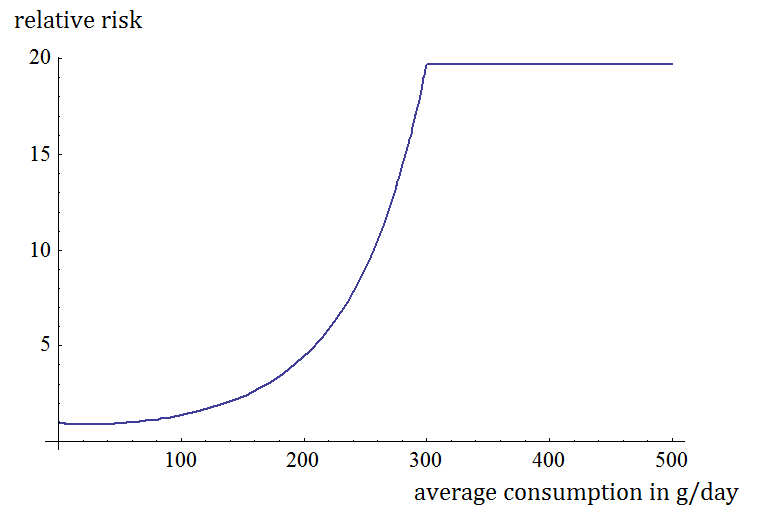


Figure 3: relative risk function of diabetes

In the case of diabetes, the function was simply capped at its value at 300 grams per day.

| **changes made on relative risk functions after 150g/day** | | |
| --- | --- | --- |
|  | **men** | **women** |
| **Oral cavity and pharynx cancer** | 200-300 g/day : linear with same average slope as between 150 and 200 g/day | 200-300 g/day : linear with same average slope as between 150 and 200 g/day |
|  | after 300g/day: capped | after 300g/day: capped |
| **oesophagus cancer** | cap at 300g/day | cap at 300g/day |
| **colon cancer** | no change | no change |
| **rectum cancer** | no change | no change |
| **liver cancer** | 200-300 g/day : linear with same average slope as between 150 and 200 g/day | 200-300 g/day : linear with same average slope as between 150 and 200 g/day |
|  | after 300g/day: capped | after 300g/day: capped |
| **larynx cancer** | 200-300 g/day : linear with same average slope as between 150 and 200 g/day | 200-300 g/day : linear with same average slope as between 150 and 200 g/day |
|  | after 300g/day: capped | after 300g/day: capped |
| **breast cancer** | no change | no change |
| **epilepsy** | no change | no change |
| **pancreatitis** | after 150g/day: linear with same average slope as between 100 and 150g/day | after 150g/day: linear with same average slope as between 100 and 150g/day |
| **lower respiratory infections** | no change | no change |
| **hemorrhagic stroke** | no change | no change |
| **ischemic stroke** | no change | cap at 300g/day |
| **hypertension** | no change | cap at 300g/day |
| **liver cirrhosis** | 200-300 g/day : linear with same average slope as between 150 and 200 g/day | 200-300 g/day : linear with same average slope as between 150 and 200 g/day |
|  | after 300g/day: capped | after 300g/day: capped |
| **diabetes** | cap at 300g/day | no change |
| **tuberculosis** | no change | no change |
| **IHD** | no change | no change |
| **MVA** | no change | no change |
| **suicide** | no change | no change |
| **other injuries** | no change | no change |
